# Identification of WTAP-related genes by weighted gene co-expression network analysis in ovarian cancer

**DOI:** 10.1186/s13048-020-00710-y

**Published:** 2020-09-30

**Authors:** Jing Wang, Jing Xu, Ke Li, Yunke Huang, Yilin Dai, Congjian Xu, Yu Kang

**Affiliations:** 1 Obstetrics and Gynecology Hospital, Fudan University, No.419, Fangxie Road, Shanghai, 200011 People’s Republic of China; 2 Shanghai Key Laboratory of Female Reproductive Endocrine Related Diseases, Shanghai, 200011 China

**Keywords:** Ovarian cancer, Progression, WTAP, WGCNA, HBS1L, FAM76A

## Abstract

**Background:**

Wilms tumor 1 associated protein (WTAP) modulates other genes via transcriptional and post-transcriptional regulation, in particular, by acting as a N6-methyladenosine writer or binding to the 3’UTR of mRNA, and promotes a variety of tumuors. However, the roles and mechanisms of WTAP in ovarian cancer are unknown.

**Results:**

In this study, using univariate Cox analysis and online CPTA analysis, we found that WTAP was a poor prognostic factor for ovarian cancer, and its protein expression level was higher in ovarian cancer than in normal tissue. Functionally, WTAP promoted the proliferation, invasion, and migration capability of ovarian cancer, according to the results of real time cellular analysis (RTCA), EdU cell proliferation assay, transwell assay. Subsequently, we identified a module containing 133 genes that were carefully related to WTAP expression through weighted gene co-expression network analysis (WGCNA). By calculating the hazard ratios of these genes and comparing their differences in the WTAP high-expression group and the low-expression group, we observed that there was a significant positive correlation between WTAP and two poor survival-related genes, family with sequence similarity 76 member A (FAM76A) and HBS1 like translational GTPase (HBS1L), which was also verified by quantitative real-time PCR in SKOV3 and A2780 cells.

**Conclusion:**

WTAP functions as an oncogenic factor that promotes the progression of ovarian cancer in which WTAP-HBS1L/FAM76A axis may be involved. Our study indicates the potential role of WTAP in prognostic biomarker and therapeutic target for ovarian cancer.

## Introduction

Ovarian cancer is the most lethal malignancy of the female reproductive tract, with a 5 year survival rate of only 29% in the advanced stage, and approximately 75% of cases are diagnosed in that stage [1]. Primary debulking surgery followed by platinum-based combination chemotherapy has become the standard treatment in advanced ovarian cancer since the 1980s [2]. However, during the past years, patients with high-grade serous ovarian cancer have experienced little improvement in overall survival, and standard treatment has not advanced beyond the previous one [3]. Therefore, investigation of the mechanisms of ovarian cancer progression is needed, which may provide insight for new treatment targets to improve ovarian cancer prognosis.

N6-methyladenosine (m6A) modification, the most common reversible post-transcriptional modification of RNA, affects cancer progression by regulating mRNA metabolism, including translation, stabilization, degradation, splicing and microRNA processing when deregulation occurs [4]. This post-translational modification is performed by methyltransferases (writer), demethylases (eraser),and m6A binding proteins (reader) [5]. As a m6A writer, Wilms tumour 1 associated Protein (WTAP) contributes to the localization of the methyltransferase METTL3-METTL14 to the nuclear speckle [6]. Other post-translational regulations that WTAP participated in include binding to the 3′-untranslated region (3’UTR) to generate increased stability of mRNA [7–9] and acting as spliceosomes to participate in alternative splicing of pre-mRNA [10]. Moreover, translational regulation is also involved in the roles of WTAP. By binding transcription factor WT1, WTAP represses transcription of WT1’s target genes, such as amphiregulin, Bcl-2 [11] and TBL1 [12].

In a previous study, WTAP-guided m6A modification was found to contributes to the progression of hepatocellular carcinoma via the HuR-ETS1-p21/p27 axis [13]. In pancreatic cancer, WTAP can bind to and stabilize Fak mRNA to activate the Fak-PI3K-AKT and Fak-Src-GRB2-Erk1/2 signalling pathways, thereby promoting metastasis and chemoresistance [8]. Moreover, WTAP may play an oncogenic role in renal cell carcinoma by binding to the CDK2 transcript and enhancing its stability [9]. Although roles and mechanisms of WTAP have been elucidated in several types of carcinoma, including colon carcinoma [12], cholangiocarcinoma [14], glioma [15], hepatocellular carcinoma [13], pancreatic carcinoma [8] and renal cell carcinoma [9], they remain elusive in ovarian cancer. It was intriguing whether WTAP also contributes to cancer progression and affects the expression of tumour-related genes through transcriptional or post-transcriptional regulation of mRNA in ovarian cancer. To determine the impact WTAP exerts in ovarian cancer, we applied bioinformatics analyses and in vitro experiments. Our results imply that WTAP promotes the malignant phenotype of ovarian cancer. Meanwhile, the joint positive correlation in the expression of WTAP-FAM76A and WTAP-HBS1L indicates the underlying mechanism which may provide new ideas for the study of ovarian cancer.

## Methods

### Cell culture and transfection

The human ovarian cancer cell lines used for the experiments, including A2780 and SKOV3, were obtained from the Shanghai Key Laboratory of Female Reproduction Endocrine Related Diseases, Obstetrics and Gynecology Hospital, Fudan University. All cells were routinely cultured with RPMI 1640 supplemented with 10% fetal bovine serum and 1% Penicillin-Streptomycin, then incubated in 5% CO2, 37 °C incubator with a humidified atmosphere.

Lentiviruses expressing shWTAP or shNC were purchased from GenePharma (China). 1 × 10^5^ cells were planted into a well of 6-well cell culture cluster and transfected with an appropriate multiplicity of infection (SKOV-3:20, A2780:5). Infected cells were selected using 1 μg/ml puromycin for 1 week. The knockdown efficiency determined by western blot assay. (sh-WTAP: GCGAAGTGTCGAATGCTTATC; sh-NC: GTTCTCCGAACGTGTCACGT).

### Western blot assay

Total protein was extracted using RIPA buffer (Beyotime, China) containing protease and phosphatase inhibitors (Beyotime, China). The BCA Protein Assay kit (Beyotime, China) was used to quantify the protein concentration. Thirty μg of each protein sample was separated by 10% SDS-PAGE gel (EpiZyme, China) and then transferred to 0.22 μm PVDF membranes (Millipore, USA). After being blocked by 5% non-fat milk in TBST for 1 h, membranes were incubated with antibody against WTAP (4A10G9, Proteintech, China) or β-Actin (8H10D10, CST, USA) at 4 °C overnight. After TBST washing for three times, the membrane were incubated with HRP-conjugated secondary antibodies for 1 h at room temperature. The immunoblots were detected following Enhanced Chemiluminescent (NCM Biotech, China).

### Real-time measurement of cell poliferation (RTCA)

The xCELLigence RTCA TP instrument (Agilent, USA) was used as described in the instruction manual. Cells (5000 cells/ well) were seeded in the E-plate (Agilent, USA) where electrodes are coated on. Each group was measured in triplicates. The cell index were measured every 15 min. As cells adhere to and proliferate on the electrodes, the flow of current is blocked, causing changes of impedance which reflect the cell proliferation activity.

### EdU proliferation assay

The 5-ethynyl-20-deoxyuridine (EdU) cell proliferation kit (Beyotime, China) was used to detect the cell proliferation ability. Cells were incubated with 10 μM EdU for 2 h, then fixed with 4% paraformaldehyde for 15 min and permeabilized with 0.1% Triton X-100 for 15 min. The click solution was added followed by the staining of nuclei with Hoechst. Images were captures by a fluorescence microscope.

### Transwell invasion and migration assay

The transfected cells (2 × 10^4^ cells/well in 200 μl serum-free culture medium) were seeded into the 24-well Matrigel-coated or uncovered upper chamber with 8-μm pore size (Corning), and 400ul medium containing 10% FBS was added to the lower chambers as a chemoattractant. After incubation for 48 h, the still cells in the upper chamber were gently scraped by cotton swabs while the invasive or migrated cells located on the lower surface of upper chambers were fixed in 4% paraformaldehyde and dyed with 0.1% crystal violet for 15 min. Each group was measured in triplicates and cells were photographed in four random fields under an inverted microscope.

### RNA extraction and quantitative real-time PCR (qRT-PCR)

Total RNAs were extracted from cultured cell lines by Trizol reagent (Invitrogen, USA). Then cDNA was synthesized using the PrimeScript™ RT Master Mix (Takara, Japan) and qRT-PCR was performed with TB Green® Premix Ex Taq™ II (TaKaRa, Japan) according to the manufacturer’s instructions. Relative RNA amount was calculated by 2^-ΔΔCt^ method with the normalization to GAPDH. The specific primers used for RT-PCR included: GAPDH, 5’GGGAAGGTGAAGGTCGGAGT 3′ (forward), 5’GGGGTCATTGATGGCAACA3’ (reverse); WTAP, 5’GTAGACCCAGCG ATCAACTTGT3’ (forward), 5’GCGTAAACTTCCAGGCACTC3’ (reverse); FAM76A, 5′ TAACTTCTAGGCGTTTCCTGG3′ (forward), 5’ACATGGTCCACTGTATCCCTT3’ (reverse); HBS1L, 5’TGATTGTGGTTGAAAGGAGTG 3’ (forward), 5′ GGCATGTCTGCTTTACCTCTT 3′ (reverse).

### Data download

The matrix of GSE63885 containing mRNA microarray data of 101 ovarian cancer samples and corresponding clinical information were downloaded from GEO. Twenty-six samples with incomplete survival information were excluded. The matrix was normalized by the R package “limma”. RNA-Seq data of 379 samples of ovarian cancer were downloaded from TCGA. The data format was HTSeq-FPKM.

### Identification of prognostic m6A regulators

In this study, we included m6A regulators including the writers METTL3, METTL14, WTAP, VIRMA, RBM15, and ZC3H13; the erasers, ALKBH5 and FTO; and the readers, HNRNPA2B1, HNRNPC, IGF2BP1, IGF2BP2, IGF2BP3, YTHDC1, YTHDC2, YTHDF1, YTHDF2, and YTHDF3. For GEO data, we applied univariate Cox analysis to identify regulators related to ovarian cancer prognosis. Genes with *P* values less than 0.05 were considered statistically significant.

### Construction of the co-expression network

The top 5000 genes with a significant median absolute deviation (MAD) in 75 ovarian cancer samples in GSE63885 were selected and qualified as an expression matrix to establish a co-expression network using the “WGCNA” package in R [16]. The clinical traits table contained the sample’s overall survival (OS), WTAP expression, grade, and stage information, which were all converted into numerical values: Figo stage (IIB,IIC: 2; IIIA, IIIB, IIIC: 3; IV:4); grade (G2: 1; G3: 2; G4: 3). For the WTAP expression, we used the function “surv_cutpoint” in the R package “survminer” to determine the best cutoff value most relevant to survival. The WTAP expression higher than the best cutoff value was marked as “2”, and the WTAP expression of samples less than the best cutoff value was marked as “1”. Another method was to directly use WTAP expression values as clinical information.

In the beginning, a hierarchical clustering tree was constructed using the expression matrix to detect outliers. Then, we calculated the correlation matrix by Pearson’s correlation analysis. Subsequently, the function “pickSoftThreshold” was used to select the optimal soft threshold β, on which the conversion from the correlation matrix into an adjacency matrix is reliant to make a scale-free co-expression network. To divide genes with similar expression patterns into different modules, the topological overlap dissimilarity measure (TOM) was used to calculate the degree of association between genes. Then, the topological overlap matrix and corresponding dissimilarity matrix were obtained. Based on the high topological overlap of genes in the module, hierarchical clustering dendrograms were constructed and the adaptive branch pruning method was used to divide and merge modules with a similar module eigengene (ME), the first principal component of a module. A minimum of 30 genes per module was required.

### Identification of the hub module and functional annotation

Correlation analysis was performed using the ME of each module and numerical clinical information, and the association was represented by the Pearson coefficient. The module highly relevant to a given clinical feature was selected for further analysis. For functional annotation, we used the R package “clusterProfiler” [17] to conduct Gene Ontology (GO) and Kyoto Encyclopedia of Genes and Genomes (KEGG) enrichment analysis. *P* values < 0.05 were considered statistically significant.

### Identification of hub genes

For genes in the hub module, we used univariate Cox analysis to screen genes that were significantly associated with prognosis. Genes with *P* values less than 0.01 were deemed the module’s prognostic genes. According to the expression level of WTAP, we used the quartile method to divide the sample into two groups: WTAP low-expression group (0–25%) and WTAP high-expression group (75–100%). The Wilcoxon test was used to compare the expression of the module’s prognostic genes between the two groups. *P* values < 0.01 were considered statistically significant.

### Gene-set enrichment analysis

Gene-set enrichment analysis (GSEA) software was downloaded from https://www.gsea-msigdb.org/gsea/datasets.jsp (Accessed 22 February 2020). Based on the median gene expression, we divided the samples into two groups and selected the C2 (c2.cp.kegg.v6.1.symbols.gmt) sub-collection downloaded from the Molecular Signatures Database https://www.gsea-msigdb.org/gsea/msigdb/collections.jsp#C2. (Accessed 22 February 2020) as the reference gene set to perform GSEA [18, 19].

### Online analysis

For online survival analysis, Kaplan-Meier Plotter was used (https://kmplot.com/analysis/index.php?p=service&cancer=ovar; Accessed 20 February 2020.) [20]. For protein analysis, we compared Clinical Proteomic Tumour Analysis Consortium (CPTAC) samples in UALCAN (http://ualcan.path.uab.edu/; Accessed 18 February 2020) [21].

## Results

### WTAP was a poor prognostic factor for ovarian cancer

**Fig. 1:**
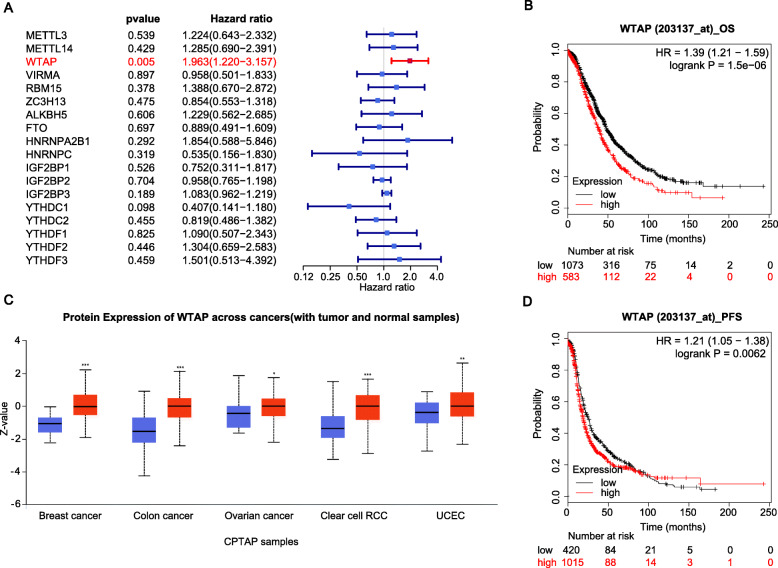
**a** Survival prognosis forest map of univariate Cox analysis in GSE63885. Each point in the forest plot represents the HR of the gene, and the line on both sides of the point represents the 95% confidence interval (95% CI). **bd** OS and PFS curves constructed by the online Kaplan-Meier plotter for ovarian cancer based on the low and high expression of WTAP. Log-rank *P* < 0.05 was considered statistically significant. **c** Protein expression of WTAP in different tumors and corresponding normal tissues obtained through CPTAC analysis in UALCAN

To investigate the effect of WTAP and other m6A regulators on the prognosis of ovarian cancer, we performed univariate Cox analysis on 18 m6A regulators. The results showed that WTAP, with the hazard ratio (HR) > 1 and *p* < 0.01 in the GSE63885 dataset, was a risk factor for ovarian cancer (Fig. 1a). At the same time, patients with high WTAP expression had dismal OS and progression-free survival (PFS) when compared with patients who had low WTAP expression in online Kaplan-Meier Plotter (Fig. 1b,d). Moreover, we noticed that protein expression of WTAP was elevated in ovarian cancer and others, including breast cancer, colon cancer, clear cell renal cell carcinoma, and uterine corpus endometrial carcinoma compared with corresponding normal tissues (Fig. 1c). We concluded that WTAP was a risk factor that negatively affects the prognosis of patients with ovarian cancer.

### WTAP promoted the malignant phenotype of ovarian cancer in vitro

**Fig. 2:**
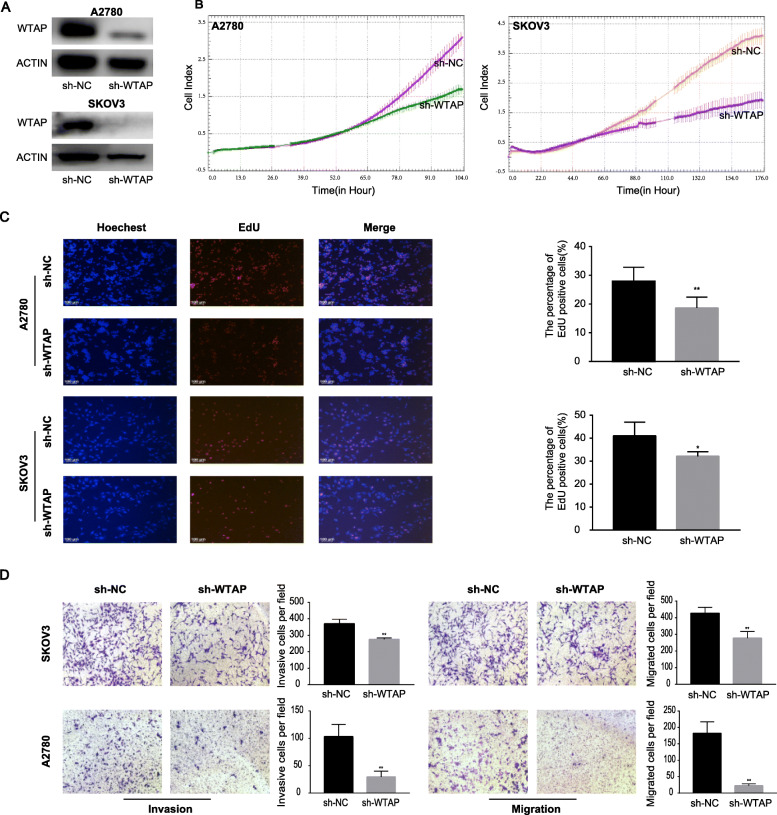
**a** Stable WTAP silenced cells (sh-WTAP) and negative control (sh-NC) were constructed by Lentiviruses. The knockdown efficiency was tested by western blot assay. **b** The cell index curves reflecting cell number were detected by RTCA. **c** EdU assay was applied to compare the cell proliferation ability. **d** The effect of WTAP knockdown on cell invasion and migration was detected by transwell assay. Statistical signifcance, as indicated by *P* values, was determined by Student’s *t*-test: *p <* 0.05(*), *p <* 0.01(**)

To explore the function of WTAP, we constructed stable WTAP-knockdown A2780 and SKOV3 cells by lentivirus infection, and the efficiency was validated by Western blot (Fig. 2a). Next, RTCA and EdU assays were carried out to investigate the impact of WTAP on cell proliferation. As reflected by cell index, cell proliferation in both A2780 and SKOV3 cell lines was significantly restrained after WTAP-knockdown (Fig. 2b). In addition, the EdU assay suggested that WTAP-knockdown cells had low EdU^+^ proportion, which indicated an attenuated proliferation ability (Fig. 2c). Furthermore, transwell assays was employed to evaluate the impact of WTAP on ovarian cancer cell motility. There was a substantial difference of cell in migration and invasion when WTAP was suppressed in both A2780 and SKOV3 cells (Fig. 2d).

### Construction of co-expression network

**Fig. 3:**
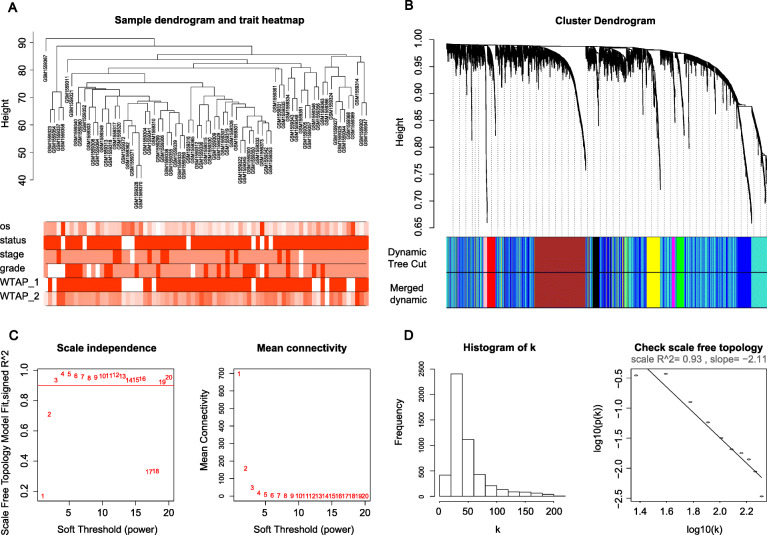
**a** Clustering of 75 ovarian cancer samples and corresponding clinical information where the numeric information is larger, the darker color is shown. **b** Cluster dendrogram in which branches correspond to gene modules based on topological overlaps and each piece of the leaves represents a gene. **c** The scale-free index and the average connectivity calculated under different β. The approximate scale-free topology can be achieved at a soft threshold power of 3. **d** Histogram of connectivity distribution and checking the scale-free topology when β = 3

As mentioned above, we selected the top 5000 genes with a significant MAD in 75 ovarian cancer samples as the expression matrix to establish a hierarchical clustering tree, and no outliers were removed (Fig. 3a). We chose β of 3 (scale-free R^2^ = 0.93) as the appropriate soft-thresholding value to ensure a scale-free network (Fig. 3c,d). Finally, a total of 9 modules were identified (Fig. 3b).

### Identification of a hub module associated with WTAP expression and functional annotation

**Fig. 4:**
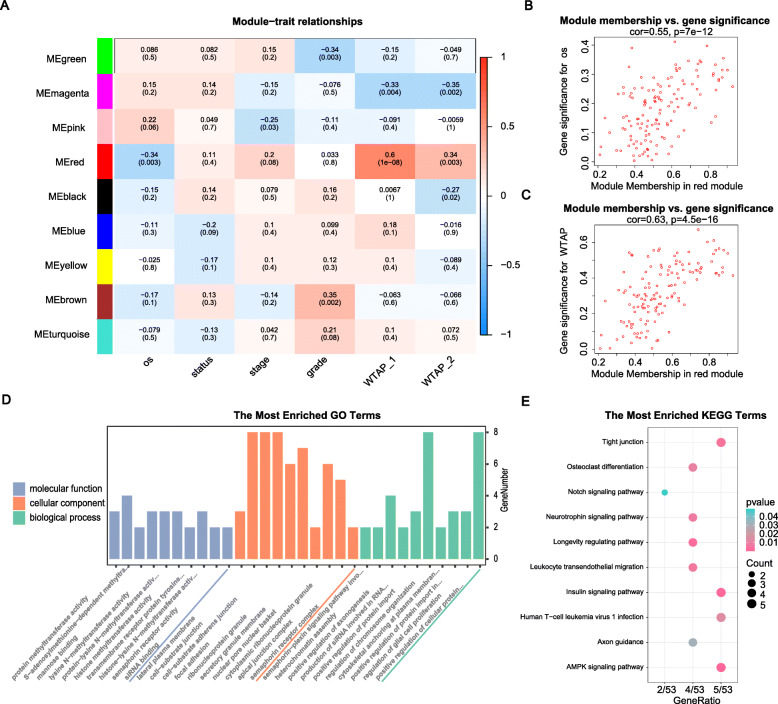
**a** Heatmap of the correlation between MEs and clinical traits. The numbers in brackets represent the *P* values, and the numbers without the brackets indicate the correlation. **bc** Correlation between MM and GS for OS or WTAP expression. The former indicates the correlation between genes and MEs, and the latter indicates correlation between genes and clinical trait. **de** GO and KEGG enrichment analysis of 133 selected genes. *P* < 0.05 was considered statistically significant

The association between the ME of each module and numerical clinical information was represented by the Pearson’s coefficient (Fig. 4a), among which the coefficient between the red module and the WTAP expression was the highest and positive (Pearson cor = 0.6, *p* = 1e-8). At the same time, the red module was also negatively correlated with OS (Pearson cor = − 0.34, *p* = 0.003). Therefore, we chose the 133 genes in the red module for further analysis. The scatter plot showed the correlation between module membership (MM; association between genes and modules) and gene significance (GS; association between genes and clinical trait) for WTAP expression or OS, respectively (Fig. 4b, c).

To explore the function of genes in the red module, we performed GO analysis. Enriched biological processes (BPs) were mainly “positive regulation of cellular protein localization”and“regulation of chromosome organization”. The cellular components (CCs) were primarily enriched in “focal adhesion”, “cell-substrate adherens junction” and “cell-substrate junction”. Enriched molecular functions (MFs) chiefly comprised “S-adenosylmethionine-dependent methyltransferase activity”(Fig. 4d). KEGG pathway analysis showed that the “AMPK signalling pathway”, “Insulin signalling pathway”, “Tight junction” and“Human T-cell leukemia virus 1 infection” pathways were the most enriched (Fig. 4e).

### Identification of hub genes that may be regulated by WTAP

**Fig. 5:**
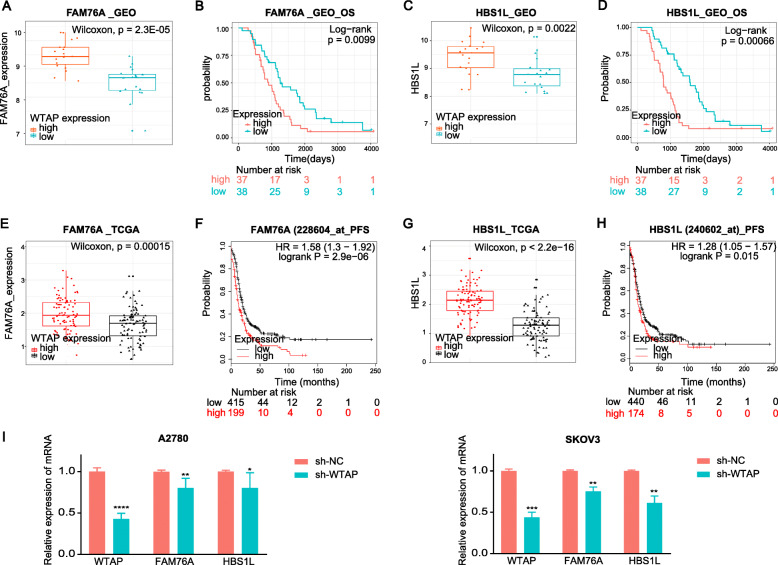
**ac** Difference of FAM76A and HBS1L expression between WTAP high-expression and low-expression expression groups in GSE63885. Wilcoxon *P* < 0.05 was considered statistically significant. **bd** OS curves of FAM76A and HBS1L in GSE63885. **eg** Difference of FAM76A and HBS1L expression between WTAP high-expression and low-expression groups in TCGA. **fh** PFS curves of FAM76A and HBS1L from the online Kaplan-Meier plotter for ovarian cancer. **i** Relative expression of mRNA in WTAP-knockdown cell and its conrol

To further determine the prognosis related genes that may be regulated by WTAP, we performed univariate Cox analysis of all genes in the red module, and the screening threshold was *p* < 0.01. Twenty genes in the red module were selected as prognostic genes for subsequent analysis (Table 1). According to the expression level of WTAP, we divided samples into two groups with quartile method: WTAP high-expression group (WTAP expression is higher than Q4) and WTAP low-expression group (WTAP expression is lower than Q1). Then, we compared the expression differences of 20 prognostic genes in the red module. Genes with significant differences (*P* < 0.01) were: FAM76A, HBS1L, MPDU1, UBE2M, PSENEN, and NRBP1 (Table 2). Among them, FAM76A and HBS1L were highly expressed in the WTAP high-expression group, and also expressed at low levels in the WTAP low-expression group (Fig. 5a, c), which can be confirmed with TCGA data (Fig. 5e, g). At the same time, the high expression of FAM76A and HBS1L also suggested poor survival in ovarian cancer patients (Fig. 5b, d, f, h). The qRT-PCR result also implied a decrease of FAM76A and HBS1L expression when WTAP was suppressed (Fig. 5i). Given the close relationship and their prognostic effects, we speculated that the mRNA of FAM76A and HBS1L may be regulated by WTAP mediated m6A modification and were affected by the positive-reader effect to promote their stability. Apart from that, the binding of WTAP to the 3’UTR may also play a role.

**Table 1:** Prognostic genes in the red module

Gene	HR	HR.95 L	HR.95H	*P* value
AGO2	2.025000718	1.374608716	2.98312375	0.000357437
UNC93B1	1.502221016	1.184893867	1.904531742	0.000775895
FAM76A	1.909579712	1.304432604	2.795464223	0.000878707
ADAM15	2.82313392	1.493892582	5.335112594	0.001393395
CLDN3	1.422241764	1.144704285	1.767069157	0.001472124
HBS1L	2.06855213	1.302189037	3.285934524	0.002082551
UBE2M	1.978033509	1.273960875	3.071221918	0.002376843
PSENEN	1.513470421	1.152289873	1.987861536	0.002892504
CLN6	2.184416433	1.29885511	3.673754769	0.003221297
HES4	1.454077184	1.122018693	1.884407516	0.004649206
MFSD14C	2.419201169	1.301407526	4.497080415	0.005225539
NRBP1	1.804336165	1.176882937	2.766315064	0.006789802
AGPAT2	1.781321775	1.16794261	2.716834921	0.007344708
MARK2	1.619059052	1.137150146	2.305194459	0.007519206
EXPH5	1.639797715	1.140602095	2.357471161	0.007579483
OAZ2	2.090922206	1.212291813	3.606355851	0.007997563
NBEAL2	1.838287134	1.166020595	2.898147426	0.008760691
MPDU1	1.765161875	1.152584527	2.703312748	0.008976294
FASN	1.581298787	1.121306047	2.229994088	0.008981118
GNB2	1.559006501	1.115853534	2.178154388	0.009257287

*P* < 0.01 was considered statistically significant

**Table 2:** Significant genes among prognostic genes in the red module when comparing expression difference in WTAP low expression group and high expression group

Gene	WTAP low-expression group	WTAP high-expression group	logFC	*P* value
FAM76A	8.516567684	9.489020421	0.155987043	2.30E-05
HBS1L	8.787158947	9.413139105	0.099279124	0.00222178
MPDU1	8.993703316	9.516729947	0.08155064	0.004175536
UBE2M	9.348555211	9.949933684	0.089943492	0.004618308
PSENEN	9.207348579	10.10675216	0.134461785	0.005629317
NRBP1	8.574702737	9.044152947	0.076898732	0.005629317

*P* < 0.01 was considered statistically significant

### Gene-set enrichment analysis of real hub genes

**Fig. 6:**
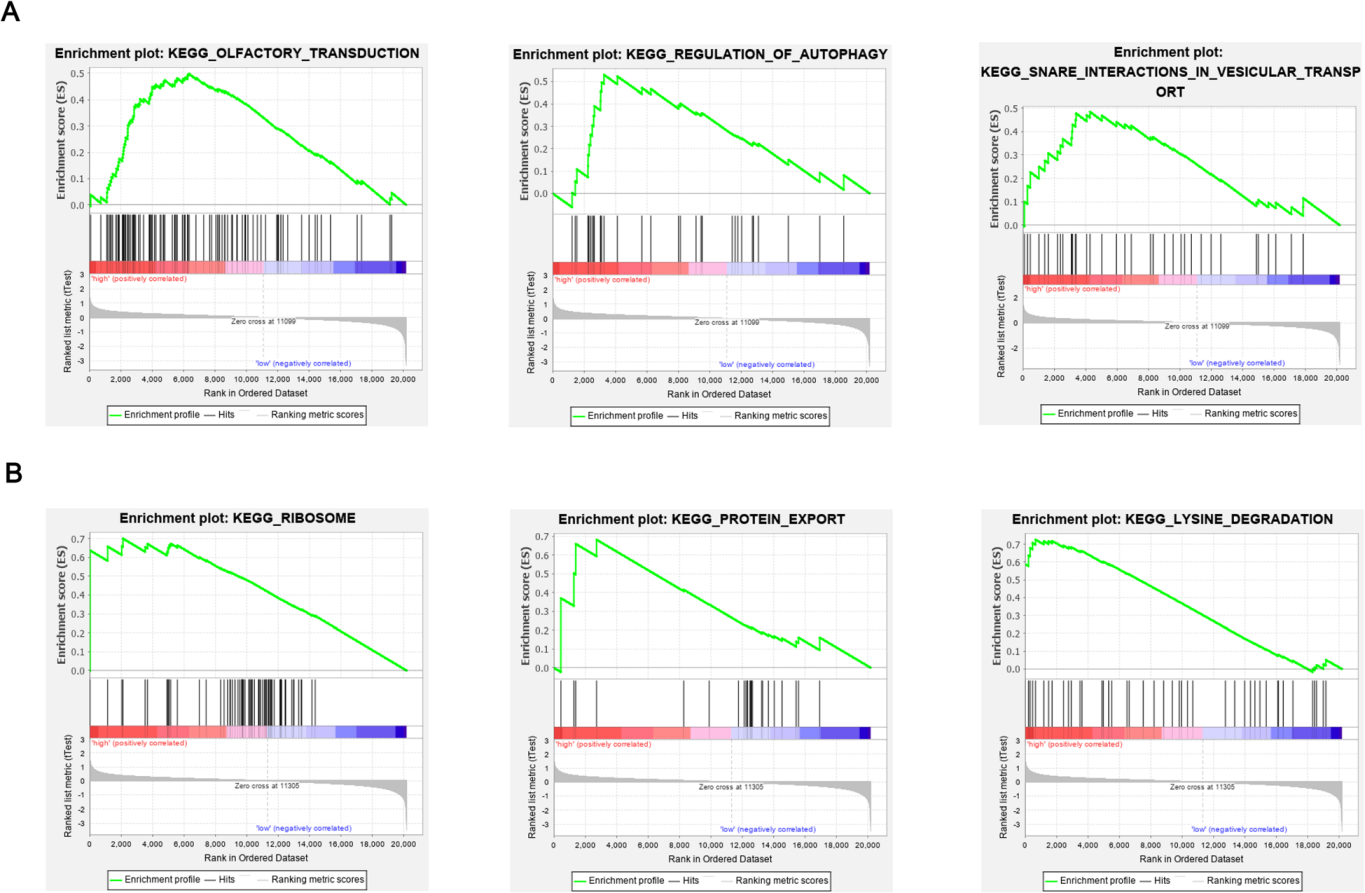
**a** Pathways highly enriched in HBS1L high-expression group through GSEA. **b** Pathways profoundly enriched in FAM76A high-expression group through GSEA

GSEA was performed to determine a potential mechanism for HBS1L and FAM76A involved in ovarian cancer. Three gene sets were enriched in the high HBS1L expression group: KEGG_OLFACTORY_TRANSDUCTION (NES = 2.11, NOM *p*-value = 0.002, FDR q-value = 0.007), KEGG_REGULATION_OF_AUTOPHAGY(NES = 1.78, NOM p-value = 0.006, FDR q-value = 0.132), and KEGG_SNARE_INTERACTIONS_IN_VESICULAR_TRANSPORT (NES = 1.73, NOM p-value = 0.008, FDR q-value = 0.152) (Fig. 6a). In the high FAM76A expression group, enriched gene sets included KEGG_RIBOSOME (NES = 1.62, NOM *p*-value = 0.04, FDR q-value = 0.152), KEGG_PROTEIN_EXPORT (NES = 1.62, NOM *p*-value = 0.018, FDR q-value = 0.076), and KEGG_LYSINE_DEGRADATION(NES = 1.6, NOM *p*-value = 0.016, FDR q-value = 0.078) (Fig. 6b).

## Discussion

In this study, we evaluated the effects of 18 regulators of m6A modification on the prognosis of ovarian cancer and found that high expression of WTAP was a risk factor for the prognosis of ovarian cancer. Subsequently, with the help of WGCNA, we identified two hub genes: FAM76A and HBS1L, which may be regulated by WTAP. The mechanisms of WTAP regulating other genes include transcriptional and post-transcriptional levels. In cancers, the latter, especially m6A modification and binding to the 3′UTR of mRNA, matters.

Mechanisms of m6A modification disorders aggravating cancer can be attributed to the following factors. First, is the target gene of m6A a cancer-promoting gene or cancer-suppressing gene? Second, is the methylation modification level of the target mRNA methylated or demethylated? Finally, is the role of m6A reader positive or negative? For example, IGF2BP1, a positive-reader that enhances mRNA stability and storage, promotes the expression of oncogene SRF in an m6A-dependent manner in ovarian cancer [22]. In endometrial cancer, METTL14 mutation or METTL3 down-regulation leads to a decrease of mTOR m6A levels, which is a regulator of oncogene AKT. YTHDF2, a negative reader promoteing m6A mRNA degradation, thus reduces degradation of mTOR and activates the AKT pathway [23]. Adenosine and uridine rich elements (AREs) are among the important cis-elements in 3’UTR. RNA-binding proteins (RBPs) combined with AREs can accelerate mRNA decay or improve its stability [24]. Similar to the mechanism mentioned above, WTAP can bind to the 3’UTR of cyclin A2 mRNA to improve its stability in umbilical vein endothelial cells. The nine-base sequence ACAAAUUAU, which is also rich in A and U, is necessary for its binding [7].

HBS1L, also known as eRF3c, encodes protein belongs to eRF3 family member which has multifunctional properties including translation termination [25], mRNA decay [26] etc. It’s reported that Polyglycine expansions in eRF3 regulating its expression and/or changing the protein function are associated with gastric cancer susceptibility [27]. Similarly, eRF3 12-GGC allele increases the susceptibility for breast cancer development [28]. The significantly positive correlation between WTAP expression and FAM76A and HBS1L in our study leads us to speculate the possibility that WTAP mediate m6A modification on the mRNA of FAM76A and HBS1L as well as coordinate with positive-readers,or WTAP bind to the 3’UTR of FAM76A and HBS1L mRNA, promoting their stability. However, how the WTAP-HBS1L/FAM76A axis plays a role in ovarian cancer progression needs further experimental verification. Another question that needs to be considered is how is the high expression of WTAP modulated? WTAP can regulate the expression of other genes, and WTAP may also be adjusted. CA4 is a tumour suppressor gene for colon cancer that inhibits the Wnt pathway by attenuating WTAP–WT1–TBL1 axis, among which WTAP is degraded because CA4 is stimulating its polyubiquitination [12]. In addition, the expression of METTL3, a “writer”similar to WTAP, is higher in gastric cancer due to p300-mediated H3K27 acetylation of the METTL3 promoter. METTL3 regulates HDGF mRNA via m6A modification, and the positive reader IGF2BP3 recognize it promoting its stability. Therefore, HDGF promotes glycolysis and angiogenesis, leading to tumuor growth and metastasis [29]. Is there a similar regulatory mechanism for WTAP expression in ovarian cancer? This question requires more researches to explain and is also our interest, which will be explored in our future work.

## Conclusions

In this study, we found that WTAP functions as an oncogenic factor that promotes the progression of ovarian cancer. Meanwhile, upregulation of the expression levels of HBS1L and FAM76A may be involved in the underlying mechanism of WTAP. WTAP may be a promising prognostic biomarker and therapeutic target for ovarian cancer.

## Data Availability

The datasets used during the current study were available from TCGA, GEO, Kaplan-Meier plotter, UALCAN that provide free online tools or open resources.

## Abbreviations

WTAP: Wilms tumour 1 associated Protein
FAM76A: Family with sequence similarity 76 member A
HBS1L: HBS1 like translational GTPase
RTCA: Real-time measurement of cell poliferation
WGCAN: Weighted gene co-expression network analysis
3’UTR: The 3′-untranslated region
m6A: N6-methyladenosine
GEO: Gene Expression Omnibus
TCGA: The Cancer Genome Atlas
OS: Overall survival
PFS: Progression- free survival
ME: Module eigengene
GO: Gene Ontology
KEGG: Kyoto Encyclopedia of Genes and Genomes
GSEA: Gene-set enrichment analysis
CPTAC: Clinical Proteomic Tumour Analysis Consortium
MM: Module membership
GS: Gene significance
AREs: Adenosine and uridine rich elements
